# Simulated Radiation Dose Reduction in Whole-Body CT on a 3rd Generation Dual-Source Scanner: An Intraindividual Comparison

**DOI:** 10.3390/diagnostics11010118

**Published:** 2021-01-13

**Authors:** Andreas S. Brendlin, Moritz T. Winkelmann, Phuong Linh Do, Vincent Schwarze, Felix Peisen, Haidara Almansour, Malte N. Bongers, Christoph P. Artzner, Jakob Weiss, Jong Hyo Kim, Ahmed E. Othman, Saif Afat

**Affiliations:** 1 Department for Diagnostic and Interventional Radiology, University Hospital Tuebingen, 72076 Tuebingen, Germany; andreas.brendlin@med.uni-tuebingen.de (A.S.B.); Moritz.Winkelmann@med.uni-tuebingen.de (M.T.W.); felix.peisen@med.uni-tuebingen.de (F.P.); haidara.al-mansour@med.uni-tuebingen.de (H.A.); Malte.Bongers@med.uni-tuebingen.de (M.N.B.); christoph.artzner@med.uni-tuebingen.de (C.P.A.); saif.afat@med.uni-tuebingen.de (S.A.); 2 Department for Cardiology, Sankt Gertrauden Hospital Berlin, 10713 Berlin, Germany; do.phuonglinh601@gmail.com; 3 Department of Radiology, University Hospital LMU, 80336 Munich, Germany; vincent.schwarze@med.uni-muenchen.de; 4 Department for Diagnostic and Interventional Radiology, University Hospital Freiburg, 79106 Freiburg, Germany; jakob.weiss.rdia@uniklinik-freiburg.de; 5 Department of Radiology, Seoul National University College of Medicine, Seoul 03080, Korea; kimjhyo@snu.ac.kr; 6 Advanced Institute of Convergence Technology, Suwon 16229, Korea

**Keywords:** CT, whole-body staging, radiation dose, 3rd generation dual-source scanner, iterative reconstruction (IR), Filtered Back Projection (FBP), Advanced Modeled Iterative Reconstruction (ADMIRE), image reconstruction

## Abstract

To evaluate the effect of radiation dose reduction on image quality and diagnostic confidence in contrast-enhanced whole-body computed tomography (WBCT) staging. We randomly selected March 2016 for retrospective inclusion of 18 consecutive patients (14 female, 60 ± 15 years) with clinically indicated WBCT staging on the same 3rd generation dual-source CT. Using low-dose simulations, we created data sets with 100, 80, 60, 40, and 20% of the original radiation dose. Each set was reconstructed using filtered back projection (FBP) and Advanced Modeled Iterative Reconstruction (ADMIRE^®^, Siemens Healthineers, Forchheim, Germany) strength 1–5, resulting in 540 datasets total. ADMIRE 2 was the reference standard for intraindividual comparison. The effective radiation dose was calculated using commercially available software. For comparison of objective image quality, noise assessments of subcutaneous adipose tissue regions were performed automatically using the software. Three radiologists blinded to the study evaluated image quality and diagnostic confidence independently on an equidistant 5-point Likert scale (1 = poor to 5 = excellent). At 100%, the effective radiation dose in our population was 13.3 ± 9.1 mSv. At 20% radiation dose, it was possible to obtain comparably low noise levels when using ADMIRE 5 (*p* = 1.000, *r* = 0.29). We identified ADMIRE 3 at 40% radiation dose (5.3 ± 3.6 mSv) as the lowest achievable radiation dose with image quality and diagnostic confidence equal to our reference standard (*p* = 1.000, *r* > 0.4). The inter-rater agreement for this result was almost perfect (ICC ≥ 0.958, 95% CI 0.909–0.983). On a 3rd generation scanner, it is feasible to maintain good subjective image quality, diagnostic confidence, and image noise in single-energy WBCT staging at dose levels as low as 40% of the original dose (5.3 ± 3.6 mSv), when using ADMIRE 3.

## 1. Introduction

Malignant diseases are the most common indication for computed tomography (CT) imaging [1]. In recent years, there has been a growing concern regarded increased radiation exposure by radiological examinations and the contribution of CT scans especially [2]. Recent data show that although the absolute number of CT scans aggregate only to approximately 9% of all radiological exams, their contribution to the patient’s total radiation exposure may be as high as 66% [3]. Since the frequency of CT examinations per person per year has risen in the last decade, managing radiation exposure according to the “as low as reasonably achievable” (ALARA) principle has become substantial in clinical routine, not least to protect organs with higher sensitivity from deterministic damages like the eye lens (max. 15 mSv/a) [3]. Nonetheless, repeated CT examinations may increase the stochastic risk of developing long-term damages like secondary malignancies even at lower doses [4]. With newer treatments and generally rising life expectancies, this still unfortunately mainly affects oncological patients who usually require regular follow-up staging CTs and are known to have elevated risks for leukemia or thyroid cancer [5]. Furthermore, previous studies have shown a significant rise in lifetime mortality in oncological patients from radiation-induced secondary malignancies [6]. Strategies for low dose CT have hence been the topic of several studies, with automated tube voltage selection and tube current modulation, as well as both low kV and low tube approaches showing promising results [7,8,9,10]. A common remaining obstacle though, is their association to significantly higher levels of image noise, potentially impairing image quality and diagnostic confidence [11,12]. Therefore, low dose CT protocols need to provide sufficient image quality to be usable for whole-body CT (WBCT) staging. In contrast to filtered back projection (FBP), the widespread adoption of iterative reconstruction (IR) algorithms has opened up a multitude of approaches for low dose CT image acquisition due to their superior noise and artifact reduction [13]. Other studies with a similar methodological approach to our study in patients with suspected cervical abscesses, and pulmonary angiography for suspected pulmonary embolisms respectively, have shown a large potential for image quality improvements at low tube acquisition in combination with iterative reconstruction techniques on 3rd generation CT scanners [14,15].

While there are many studies pertaining to low dose CT imaging, systematic intra-individual studies investigating the dose reduction potential are lacking. Therefore, we aimed to systematically compare intraindividual WBCT staging on a 3rd generation dual-source scanner and to evaluate the effects of simulated low tube acquisition in combination with the use of FBP, as well as the statistical iterative image reconstruction algorithm ADMIRE^®^ (Advanced Modeled Iterative Reconstruction Algorithm, introduced by Siemens Healthineers in late 2013) on low-contrast detectability, CT image quality and diagnostic confidence [13].

## 2. Materials and Methods

### 2.1. Population

The institutional ethic committee of university hospital of Tuebingen approved this study (approval number 414/2017BO2) and waived the requirement for informed consent. We randomly chose March 2016 as the inclusion window and selected 30 consecutive patients. Out of these, we included 18 patients (mean age 60 ± 15 years, 14 female) with clinically indicated WBCT staging. We collected biometrical details such as height and weight at the time of the examination from our clinical information system and calculated body-mass-index (BMI) values for comparison. For homogeneity of data, reasons for exclusion (*n* = 12) were dual-energy imaging, no contrast-enhanced image acquisition, and patient age <18 years.

### 2.2. Radiation Exposure

We collected radiation exposure values from the patient protocols, particularly the dose length product (DLP), the CT volume dose index (CTDI_vol_), the tube voltage (kV), and tube current-time product (mAs). We determined the total effective radiation dose using the commercial software Radimetrics (ver. 2.9, Bayer Medical Care, Leverkusen, Germany), a certified dose tracking and management tool implementing the weighting factors proposed in the International Commission on Radiological Protection (ICRP) Publication 103 [16].

### 2.3. Image Acquisition Protocol

Every staging CT was performed on the same 3rd-generation dual-source CT Scanner (SOMATOM FORCE, Siemens Healthineers, Forchheim, Germany), using a stellar detector (StellarInfinity) with improved spatial resolution, image quality, and dose efficiency [17]. We employed our institute’s standard single-energy WBCT staging protocol (base of the skull–middle femur) with attenuation-based tube current modulation (CARE Dose4D, reference mAs 180) and automatic tube voltage selection (80–120 kV, reference kV 110). For WBCT staging, matrix size was set to 512, the field of view was 50 cm, collimation was 0.6 × 192 × 3.0, gantry rotation time was 0.5 s, pitch factor was 0.6, and slice thickness, as well as increment were set to 3 mm. For scanning, patients were positioned on their backs feet-first with arms raised above their head. A contrast agent (Imerone 400; Bracco, Milan, Italy) was applied with adaption to body weight (body weight in g + 15 = amount of contrast agent in mL) through a peripheral venous cannula using an automated power injector at a flow rate of 2.2 mL/s (CT Stellant, Medrad, Indianola, PA, USA) followed by a chaser of 50 mL saline. Scanning was performed during the portal venous phase.

### 2.4. Image Reconstruction Parameters

**Figure 1:**
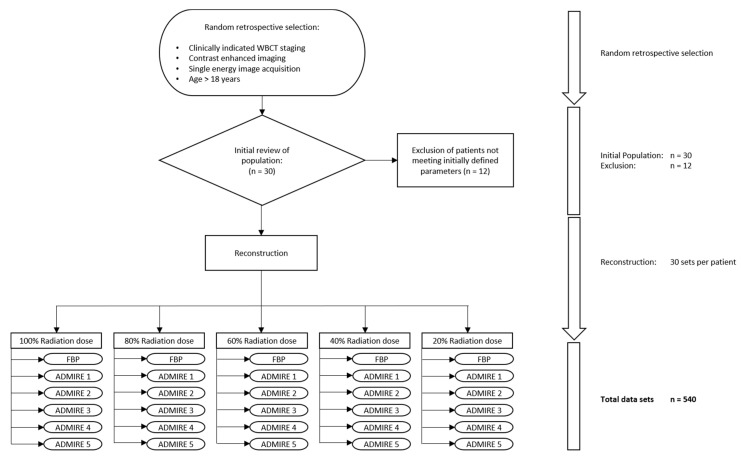
Flowchart of the patient enrollment and reconstruction process.

For reconstruction, we used the software solution ReconCT ver. 14.2.0.40998 (Siemens Healthineers, Forchheim, Germany), that additionally allows for seamless low dose simulation by adding overall image noise relative to a percentual radiation dose reduction [15]. To facilitate reading and intraindividual comparison, we chose to generate datasets with the visual impression of image noise at 100, 80, 60, 40, and 20% radiation dose. At these five simulated dose levels, axial, coronal, and sagittal reformations were reconstructed from the raw data using a medium soft kernel (Bf36d) and employing filtered back projection (FBP), as well as Advanced Modeled Iterative Reconstruction (ADMIRE^®^, Siemens Healthineers, Forchheim, Germany) strength 1, 2, 3, 4, and 5, resulting in a total of 540 datasets (30 reconstructions per patient). The computation-intensive ADMIRE algorithm processes the raw data multiple times to reduce image noise and improve low-contrast detectability, as opposed to the more traditional FBP algorithm, which processes the raw data only once. This results in an average dose reduction potential of 41% for ADMIRE reconstructions [13]. Figure 1 illustrates a detailed flow chart of the image acquisition and reconstruction process.

Since our institute uses ADMIRE 2 as the standard reconstruction method for single-energy WBCT staging in clinical routine, we defined 100% radiation dose ADMIRE 2 as the reference standard for intraindividual comparison.

### 2.5. Objective Analysis of Image Quality

**Figure 2:**
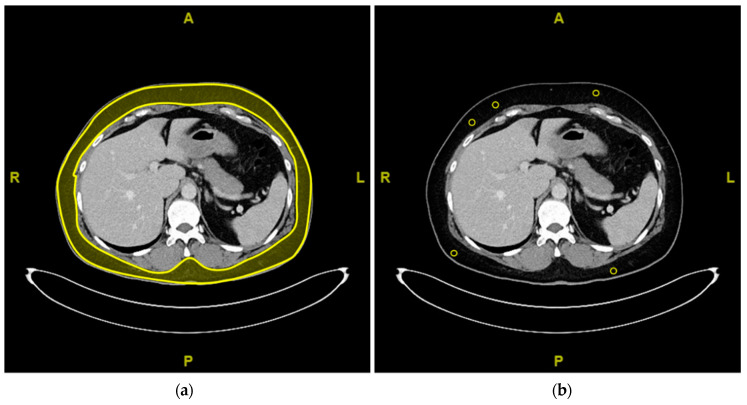
Simplified scheme of automated identification of subcutaneous adipose tissue (**a**) and random selection of 5 homogenous regions of interest (ROIs) (**b**) by the previously established algorithm [18].

Image noise was used for comparison of objective image quality. Image noise was defined as the standard deviation of Hounsfield units (HU) taken from regions of interest (ROI) placed in homogenous subcutaneous adipose tissue to account for comparability. To further limit potential mistakes during measurements, identification of average noise estimates was performed automatically by a previously established algorithm [18]. This algorithm automatically identifies subcutaneous adipose tissue, randomly selects five ROIs with 1 cm² circumference and high levels of anatomical coherence, and estimates their average noise levels. Figure 2 is a simplified scheme of the algorithm’s workflow.

### 2.6. Subjective Analysis of Image Quality

To minimize recall bias, three radiologists (AB, FP, SA) with different levels of experience level (3–8 years) independently evaluated the anonymized CT datasets in batches of 30 regarding image quality and diagnostic confidence with 6 weeks between each block of reading. To ensure blinded reading, a fourth radiologist (MW) randomized the CT datasets in advance and kept the technical background information hidden. For the subjective analysis, the CT scans were primarily displayed in a soft tissue window setting (Center 50 HU, Width 350 HU). Individual window adjustment was allowed. We rated two different regions (neck/thorax, abdomen) using the European Guidelines on Quality Criteria in Computed Tomography and chose to focus on one criterion for soft tissue and parenchymal structures, and one criterion for skeletal structures in each region respectively [19]. The analysis of the neck/thorax thus had to consider the visually sharp reproduction of the cervical and thoracic spine and the anterior mediastinal structures. For the abdomen, the criteria were a visually sharp reproduction of the lumbar spine, as well as the liver parenchyma with intrahepatic vessels. An equidistant 5-point Likert scale was used (5 = excellent, 4 = good, 3 = average, 2 = below average, 1 = poor). Grading for diagnostic confidence was performed in the same fashion. To facilitate comparison, we chose to use one grade for overall image quality and one grade for overall diagnostic confidence, each representing the median of the individual regional results.

### 2.7. Statistical Analysis

We used IBM^®^ SPSS^®^ Statistics Version 25.0.0.1 for Windows (Armonk, NY, USA) for statistical analysis. The normality of the data was tested using a Shapiro-Wilk test. Normally distributed values were given as mean ± standard deviation, non-normally distributed values as median and range. For inter-rater-agreement, an intraclass correlation coefficient (ICC) was calculated [20]. ICC values of 0–0.2 were considered as slight, 0.21–0.4 as fair, 0.41–0.6 as moderate, 0.61–0.8 as substantial, and 0.81–1.00 as almost perfect levels of agreement.

For comparison of qualitative reading scores and quantitative assessments, further statistical testing followed using ANOVA for normally distributed values, or the Friedman test for non-normally distributed values, respectively. post-hoc Dunn-Bonferroni tests ensued with proper alpha correction. Thus, a *p*-value ≤ 0.05 could be considered statistically significant. We further calculated Pearson’s r as a measure for effect size and defined *r*-values from 0.1 to 0.3 as small, 0.3 to 0.7 as medium, and ≥0.7 as large effect size.

## 3. Results

### 3.1. Population and Radiation Dose

At 100% radiation dose, we estimated a mean effective radiation dose (ED) of 13.3 ± 9.1 mSv. We calculated ED values for 80% radiation dose to be at 10.7 ± 7.2 mSv, and for 60% radiation dose at 8.3 ± 5.7 mSv. At 40% radiation dose, ED was calculated to be at 5.3 ± 3.6 mSv, and 20% of the original radiation dose levels at 2.7 ± 1.8 mSv. Table 1 shows an overview of our study population and estimated ED.

### 3.2. Objective Analysis of Image Quality

Table 2 shows the measured noise values at the different radiation dose percentages and reconstructions. There were significant interactions between the noise levels (Friedman-Test: Chi-Square (29) = 505.97, *p* < 0.001).

**Figure 3:**
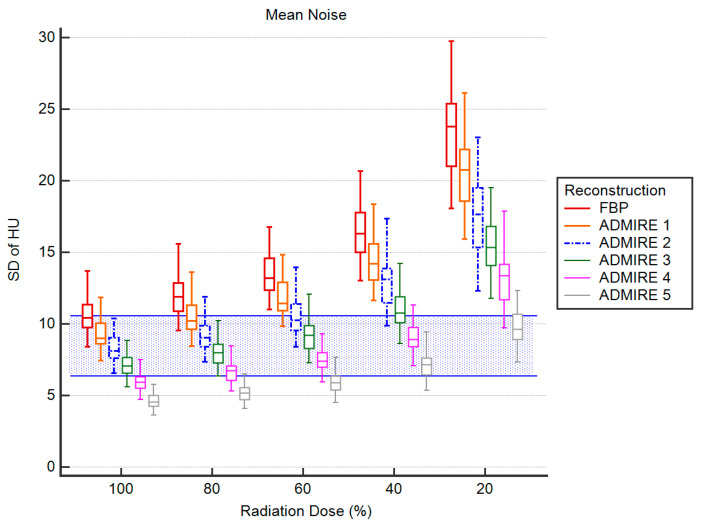
Noise Values, blue background shows the range of 100% ADMIRE 2.

There were no significant differences between the measured noise values of the reconstructions (*p* > 0.215) at 100% radiation dose. In comparison to 100% ADMIRE 2, there were also no significant differences to the noise levels measured at 80% radiation dose (*p* ≥ 0.728). At 60% radiation dose, only FBP yielded significantly higher noise values (*p* ≥ 0.007) with effect size indicating a strong effect (*r* > 0.7). At 40% radiation dose, only ADMIRE 1 and FBP showed significantly higher values of image noise (*p* ≤ 0.002; *r* > 0.7), while at 20% radiation dose, every group but ADMIRE 5 yielded significantly higher noise values (*p* ≤ 0.033; *r* > 0.7). Figure 3 is a visualization of the mean image noise levels of the different combinations of reconstruction methods and simulated radiation dose.

### 3.3. Subjective Analysis of Image Quality

#### 3.3.1. Overall Image Quality

There were significant interactions between the subjective ratings of the overall image quality (Friedman-Test: Chi-Square (29) = 485.04, *p* < 0.001). Reducing radiation dose to 60% resulted in good overall image quality (4; overall IQR 3-5) for ADMIRE 1, 2, 3, 4, and 5. Only FBP achieved significantly lower grades (*p* = 0.003, *r* > 0.7). At 40% radiation dose, ADMIRE 3 managed to perform comparably well (4; IQR 4–5) with no significant differences to our reference standard, the other groups were rated significantly lower (*p* > 0.001, *r* > 0.7). The overall image quality at 20% radiation dose generally received significantly lower grades (*p* < 0.001, *r* > 0.7) in all groups. Inter-rater-agreement concerning the overall image quality was substantial for 100% radiation dose ADMIRE 5 (ICC = 0.750, 95% CI 0.457–0.899) and almost perfect for all other groups (ICC ≥ 0.885, 95% CI 0.748–0.953). For further details, see Table 3.

#### 3.3.2. Overall Diagnostic Confidence

There were significant interactions between the subjective ratings of the overall diagnostic confidence (Friedman-Test: Chi-Square (29) = 474.67, *p* < 0.001). As with overall image quality, diagnostic confidence was rated average (3; IQR 3–4) at 60% radiation dose for FBP, ranking significantly lower (*p* = 0.002, *r* > 0.7) than the other groups. In concordance with our previous results, overall diagnostic confidence at 40% radiation dose was only comparably good (4; IQR 4–5) in ADMIRE 3 (*p* = 1.000). The other groups received significantly lower grades (*p* < 0.002, *r* > 0.7). At 20% radiation dose, overall diagnostic confidence dose was rated significantly lower (*p* < 0.001, *r* > 0.7) in all groups when compared to 100% ADMIRE 2. Inter-rater-agreement concerning the diagnostic confidence was almost perfect in all groups (ICC ≥ 0.915, 95% CI 0.814–0.966). See Table 4 for further details.

### 3.4. Images

**Figure 4:**
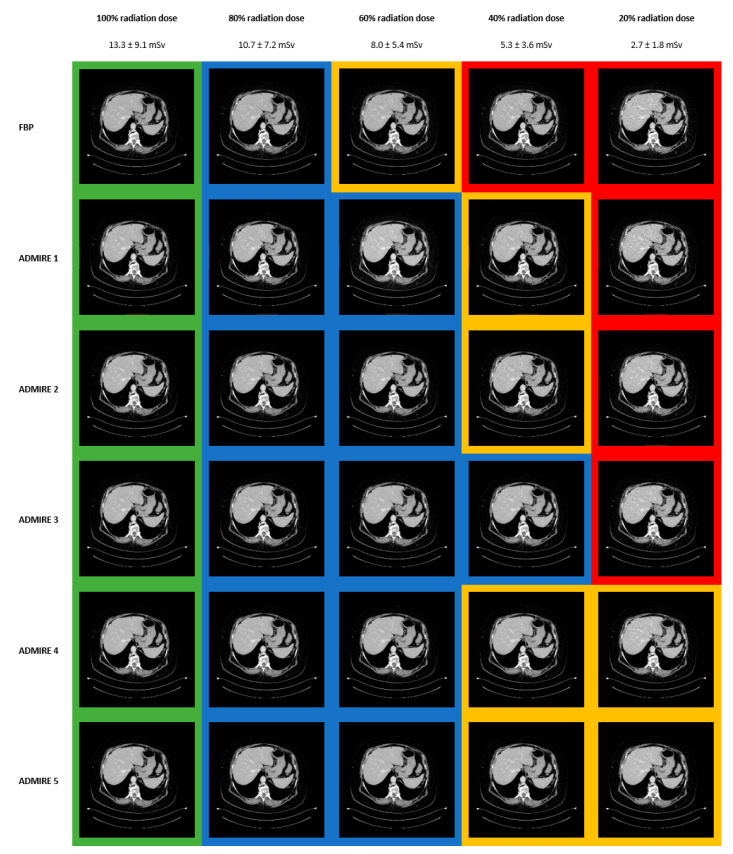
Overall Image Quality and Diagnostic Confidence.

Figure 4 shows an overview of the reconstructions at different radiation dose levels. The green background marks excellent levels of overall image quality and diagnostic confidence (identical in this patient), the blue background is good, the yellow background average, and the red background indicates below-average levels.

**Figure 5:**
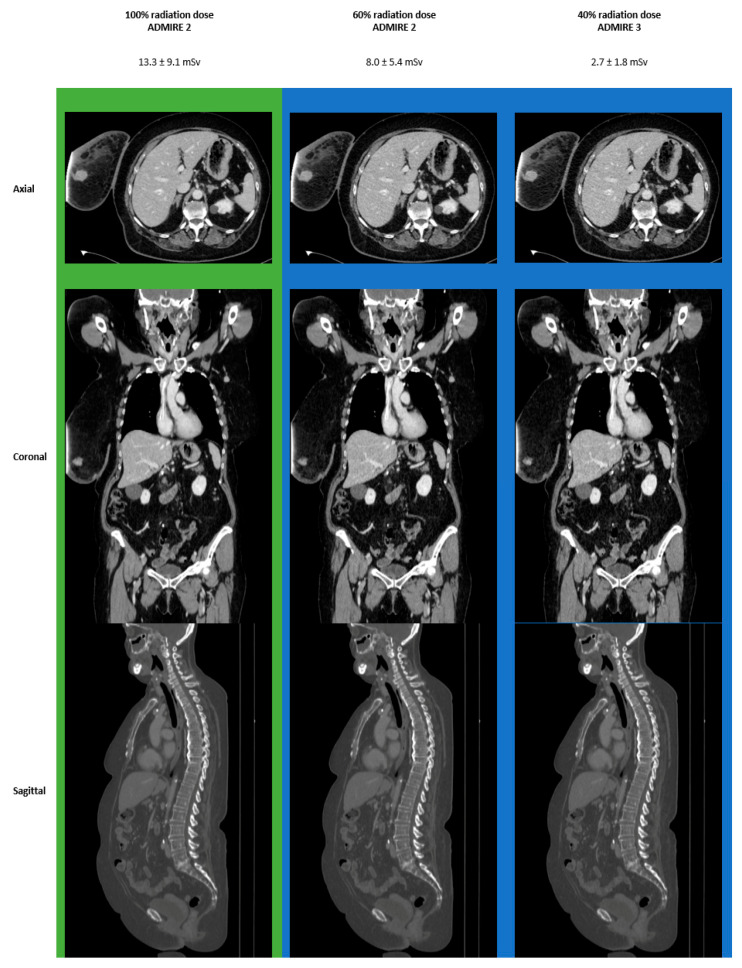
Direct Comparison 100% ADMIRE 2 vs. 60% ADMIRE 2 vs. 40% ADMIRE 3.

Figure 5 shows 100% ADMIRE 2 in direct comparison to 60% ADMIRE 2 and 40% ADMIRE 3 in a patient with an initial diagnosis of breast cancer on the right side. The green background marks excellent levels of image quality and diagnostic confidence (identical in this patient), the blue background indicates good levels.

## 4. Discussion

In this study, we systematically compared and evaluated intraindividual WBCT staging regarding the effects of tube current reduction on image noise, subjective image quality, and diagnostic confidence. We conducted this study by comparing simulated low dose WBCT at different reconstruction settings to our reference standard (100% ADMIRE 2). In the first step, we investigated image noise only to identify the lowest possible combination of radiation dose and reconstruction setting with noise levels comparable to our reference standard. At 40% radiation dose, ADMIRE 2–5 showed comparably low noise levels, and at 20% radiation dose, only ADMIRE 5 was comparable. Next, we compared the subjective ratings for image quality and diagnostic confidence, to identify the lowest possible combination of radiation dose and reconstruction setting with grades comparable to 100% ADMIRE 2. We found the lowest possible combination to be 40% ADMIRE 3, which we identified as our sweet spot.

Many recent studies showed that increasing image noise is a common problem when reducing radiation dose in CT imaging [21]. In concordance to Gordic et al., we found noise levels to decrease with higher levels of IR [22]. Another study by Scholz et al. reported a possible radiation dose reduction of up to 63% in contrast-enhanced head and neck CT at comparable noise levels when using a combination of automated dose modulation and iterative reconstruction [23]. When looking at image noise only, our findings mirror these results: our data suggest that a radiation dose reduction to 20% (2.7 ± 1.8 mSv) is feasible when choosing ADMIRE 5 without any significant differences to noise levels at 100% ADMIRE 2. Noise levels, however, can only be used to provide an impression of objective image quality, without addressing subjective image quality. In recent years, several studies have shown that a significant reduction in radiation dose is possible while maintaining good image quality when combining low dose image acquisition with iterative reconstruction [24,25,26,27]. Murphy et al. reported a possible mSv target as low as 1.05 ± 0.17 mSv for thoracic CT and 1.92 ± 0.57 mSv for abdominopelvic CT respectively at equal levels of image quality in their feasibility study regarding low dose follow-up CT image acquisition in patients with testicular cancer [28]. In comparison to our data, their target is approximately 2 mSv lower than ours. However, when reviewing and discussing our data in an unblinded reference standard assessment, our readers reaffirmed their decisions regarding subjective image quality and diagnostic confidence. In our view, the visual impression of higher IR levels at lower radiation doses was simply too overdriven and artificial to be awarded higher grades. Additionally, as some studies have pointed out before, the nonlinear smoothing of higher IR levels may even lead to a change of appearance in anatomical features at lower doses, potentially endangering diagnostic confidence and leading to wrong diagnoses [29,30,31,32]. Our scope was to identify a potential target that reliably produces images with the overall image quality of 100% ADMIRE 2 and equally high levels of diagnostic confidence. In summary, we therefore cautiously advise against targeting 2.7 ± 1.8 mSv in combination with ADMIRE 5, even though noise levels are comparable. In comparison to 100% ADMIRE 2, our findings indicate that a tube current reduction to 40% of the original radiation dose (5.3 ± 3.6 mSv) in combination with ADMIRE 3 is the sweet spot, maintaining good overall image quality and diagnostic confidence, as well as comparably low noise levels.

## 5. Limitations

Our study has several limitations. Although ReconCT software is proven to provide reliable simulations of low dose CT images, our data sets are based on simulations rather than multiple scans to avoid excessive radiation exposure. Additionally, while ReconCT allows for seamless low dose simulation from 100 to 1% radiation dose, we only focused on datasets with the visual impression of overall image noise at 100%, 80%, 60%, 40%, and 20% radiation dose. While this choice certainly facilitated reading and intraindividual comparison, more steps might have helped to narrow the target down even further. Furthermore, we chose a retrospective approach with a relatively small patient population. However, we bring forward the argument that the inherently higher statistical power of intraindividual comparisons minimizes potential biases. Indeed, a prospective study with real low dose image acquisition is needed to confirm our results. Lastly, we need to point out that this study was performed, employing our institute’s custom WBCT single-energy protocol in combination with the body-weight adapted application of Imerone 400 on a high-end 3rd generation CT scanner (Siemens SOMATOM FORCE) that is not readily available at every clinical site. Our results may therefore be specific to our setup and might not necessarily be applicable to other sites, or older scanner generations.

## 6. Conclusions

On a 3rd generation scanner, it is feasible to maintain good subjective image quality, diagnostic confidence, and image noise in single-energy WBCT staging at dose levels as low as 40% of the original dose (5.3 ± 3.6 mSv), when using ADMIRE 3.

## Figures and Tables

**Table 1:** Study Population and Radiation Dose.

Parameter		Male	Female	Total
Patient Population				
	Absolute (*n*)	4	14	18
	Reconstructions (*n*)	120	420	540
	Mean age (years)	62 ± 25	60 ± 14	60 ± 15
	Mean BMI (kg/m²)	26 ± 2	30 ± 7	30 ± 6
Diagnosis (*n*)				
	breast cancer		11	11
	melanoma	2	1	3
	squamos cell carcinoma	1	1	2
	oropharyngeal carcinoma	1		1
	lymphoma		1	1
Image Acquisistion parameters				
	kV	100.00 ± 00.0	115.71 ± 22.08	112.22 ± 20.52
	mAs	183.25 ± 16.01	218.43 ± 50.51	210.61 ± 47.19
	Mean CTDI_vol_ (mGy)	7.37 ± 0.65	15.03 ± 10.52	13.33 ± 9.8
	Mean DLP (mGy*cm)	570.85 ± 59.85	1229.8 ± 905.42	1083.37 ± 840.93
Mean Estimated Effective Radiation Dose (mSv)				
	100%	7.9 ± 1.4	14.9 ± 9.8	13.3 ± 9.1
	80%	6.3 ± 1.1	11.9 ± 7.8	10.7 ± 7.2
	60%	4.7 ± 0.8	8.9 ± 5.9	8.0 ± 5.4
	40%	3.2 ± 0.6	6.0 ± 3.9	5.3 ± 3.6
	20%	1.6 ± 0.3	3.0 ± 2.0	2.7 ± 1.8

BMI = body-mass-index; CTDI_vol_ = CT volume dose index; DLP = dose length product.

**Table 2:** Noise Values at Radiation Dose Levels.

Radiation Dose (%)	ED (mSv)	Reconstruction	Noise (SD of HU)	*p*	*r*
				vs. 100% ADMIRE 2	
100	13.3 ± 9.1	FBP	10.51 ± 1.30	1.000	0.44
		ADMIRE 1	9.22 ± 1.12	1.000	<0.1
		ADMIRE 2	8.98 ± 2.62		
		ADMIRE 3	7.06 ± 0.85	1.000	0.38
		ADMIRE 4	6.14 ± 1.25	1.000	0.58
		ADMIRE 5	4.62 ± 0.58	0.215	>0.7
80	10.7 ± 7.2	FBP	11.92 ± 1.53	1.000	>0.7
		ADMIRE 1	10.46 ± 1.33	1.000	0.42
		ADMIRE 2	9.18 ± 1.31	1.000	<0.1
		ADMIRE 3	7.95 ± 1.02	1.000	0.18
		ADMIRE 4	6.88 ± 1.38	1.000	0.46
		ADMIRE 5	5.14 ± 0.66	0.728	>0.7
60	8.0 ± 4.5	FBP	13.74 ± 1.86	0.007	>0.7
		ADMIRE 1	12.08 ± 1.63	0.491	>0.7
		ADMIRE 2	10.55 ± 1.61	1.000	0.46
		ADMIRE 3	9.18 ± 1.24	1.000	<0.1
		ADMIRE 4	7.92 ± 1.65	1.000	0.21
		ADMIRE 5	5.86 ± 0.80	1.000	0.62
40	5.3 ± 3.6	FBP	16.78 ± 2.37	<0.001	>0.7
		ADMIRE 1	14.65 ± 2.18	0.002	>0.7
		ADMIRE 2	12.95 ± 2.09	0.090	>0.7
		ADMIRE 3	11.27 ± 1.73	1.000	0.60
		ADMIRE 4	9.58 ± 2.02	1.000	0.19
		ADMIRE 5	7.00 ± 1.09	1.000	0.40
20	2.7 ± 1.8	FBP	23.85 ± 3.61	<0.001	>0.7
		ADMIRE 1	20.97 ± 3.14	<0.001	>0.7
		ADMIRE 2	18.33 ± 3.39	<0.001	> 0.7
		ADMIRE 3	15.85 ± 2.45	<0.001	>0.7
		ADMIRE 4	13.55 ± 2.85	0.033	>0.7
		ADMIRE 5	9.98 ± 1.53	1.000	0.29

ED = Effective Radiation Dose; SD = Standard Deviation; HU = Hounsfield Units.

**Table 3:** Overall Image Quality.

Radiation Dose (%)	ED (mSv)	Reconstruction	Rating		ICC	ICC: 95% CI			*p*	*r*
			Median	IQR	Average Measure	Lower Bound		Upper Bound	vs. 100% ADMIRE 2	
100	13.3 ± 9.1	FBP	5	4–5	0.942	0.874	–	0.976	1.000	<0.1
		ADMIRE 1	5	4–5	0.896	0.775	–	0.958	1.000	<0.1
		ADMIRE 2	5	4–5	1.000					
		ADMIRE 3	5	4–5	1.000				1.000	<0.1
		ADMIRE 4	5	4–5	0.896	0.775	–	0.958	1.000	<0.1
		ADMIRE 5	5	4–5	0.750	0.457	–	0.899	1.000	<0.1
80	10.7 ± 7.2	FBP	4	3–5	0.983	0.962	–	0.993	0.971	>0.7
		ADMIRE 1	4	3–5	0.980	0.957	–	0.992	1.000	0.68
		ADMIRE 2	4	4–5	0.968	0.930	–	0.987	1.000	0.48
		ADMIRE 3	4	4–5	0.970	0.935	–	0.988	1.000	0.46
		ADMIRE 4	4	3–5	0.950	0.891	–	0.980	1.000	0.60
		ADMIRE 5	4	3–5	0.956	0.904	–	0.982	1.000	0.68
60	8.0 ± 4.5	FBP	3	3–4	0.968	0.930	–	0.987	0.003	>0.7
		ADMIRE 1	4	3–4	0.961	0.916	–	0.984	0.542	>0.7
		ADMIRE 2	4	4–5	0.949	0.889	–	0.979	1.000	0.56
		ADMIRE 3	4	4–5	0.958	0.909	–	0.983	1.000	0.54
		ADMIRE 4	4	3–4	0.885	0.748	–	0.953	1.000	0.68
		ADMIRE 5	4	3–4	0.907	0.798	–	0.962	0.542	>0.7
40	5.3 ± 3.6	FBP	2	1–2	0.968	0.930	–	0.987	<0.001	>0.7
		ADMIRE 1	3	2–3	0.961	0.916	–	0.984	<0.001	>0.7
		ADMIRE 2	3	3–4	0.949	0.889	–	0.979	<0.001	>0.7
		ADMIRE 3	4	4–5	0.958	0.909	–	0.983	1.000	0.46
		ADMIRE 4	3	2–3	0.885	0.748	–	0.953	<0.001	>0.7
		ADMIRE 5	3	2–3	0.907	0.798	–	0.962	<0.001	>0.7
20	2.7 ± 1.8	FBP	2	1–2	0.974	0.943	–	0.989	<0.001	>0.7
		ADMIRE 1	2	1–2	0.971	0.938	–	0.988	<0.001	>0.7
		ADMIRE 2	2	1–2	0.968	0.930	–	0.987	<0.001	>0.7
		ADMIRE 3	2	1–3	0.968	0.930	–	0.987	<0.001	>0.7
		ADMIRE 4	3	2–3	0.933	0.853	–	0.973	<0.001	>0.7
		ADMIRE 5	3	2–3	0.936	0.861	–	0.974	<0.001	>0.7

ED = Effective Radiation Dose.

**Table 4:** Overall Diagnostic Confidence.

Radiation Dose (%)	ED (mSv)	Reconstruction	Rating		ICC	ICC: 95% CI			*p*	*r*
			Median	IQR	Average Measure	Lower Bound		Upper Bound	vs. 100% ADMIRE 2	
100	13.3 ± 9.1	FBP	5	4–5	0.958	0.909	–	0.983	1.000	<0.1
		ADMIRE 1	5	4–5	0.942	0.874	–	0.976	1.000	<0.1
		ADMIRE 2	5	4–5	1.000					
		ADMIRE 3	5	4–5	1.000				1.000	<0.1
		ADMIRE 4	5	4–5	0.942	0.874	–	0.976	1.000	<0.1
		ADMIRE 5	5	4–5	0.919	0.823	–	0.967	1.000	<0.1
80	10.7 ± 7.2	FBP	4	3–5	0.985	0.969	–	0.994	0.777	>0.7
		ADMIRE 1	4	3–5	0.984	0.966	–	0.994	1.000	0.70
		ADMIRE 2	4	4–5	0.974	0.943	–	0.989	1.000	0.37
		ADMIRE 3	4	4–5	0.973	0.941	–	0.989	1.000	0.40
		ADMIRE 4	4	3–5	0.959	0.910	–	0.983	1.000	0.59
		ADMIRE 5	4	3–5	0.962	0.918	–	0.985	1.000	0.67
60	8.0 ± 4.5	FBP	3	3–4	0.974	0.943	–	0.989	0.002	>0.7
		ADMIRE 1	4	3–4	0.971	0.823	–	0.967	0.429	>0.7
		ADMIRE 2	4	4–5	0.968	0.930	–	0.987	1.000	0.45
		ADMIRE 3	4	4–5	0.966	0.926	–	0.986	1.000	0.48
		ADMIRE 4	4	3–4	0.915	0.814	–	0.966	1.000	0.67
		ADMIRE 5	4	3–4	0.926	0.839	–	0.970	0.639	>0.7
40	5.3 ± 3.6	FBP	2	1–2	0.974	0.943	–	0.989	<0.001	>0.7
		ADMIRE 1	3	2–3	0.971	0.938	–	0.988	<0.001	>0.7
		ADMIRE 2	3	3–4	0.968	0.930	–	0.987	0.002	>0.7
		ADMIRE 3	4	3–4	0.966	0.926	–	0.986	1.000	0.40
		ADMIRE 4	3	2–3	0.915	0.814	–	0.966	<0.001	>0.7
		ADMIRE 5	3	2–3	0.926	0.839	–	0.970	<0.001	>0.7
20	2.7 ± 1.8	FBP	2	1–2	0.975	0.947	–	0.990	<0.001	>0.7
		ADMIRE 1	2	1–2	0.975	0.945	–	0.990	<0.001	>0.7
		ADMIRE 2	2	1–2	0.974	0.943	–	0.989	<0.001	>0.7
		ADMIRE 3	2	1–3	0.974	0.943	–	0.989	<0.001	>0.7
		ADMIRE 4	3	2–3	0.945	0.879	–	0.978	<0.001	>0.7
		ADMIRE 5	3	2–3	0.946	0.882	–	0.978	<0.001	>0.7

ED = Effective Radiation Dose

## Data Availability

Data sharing not applicable.
